# Factors to consider when interrogating 3D culture models with plate readers or automated microscopes

**DOI:** 10.1007/s11626-020-00537-3

**Published:** 2021-02-09

**Authors:** Terry Riss, O. Joseph Trask

**Affiliations:** 1 Promega Corporation, Cell Health, 2800 Woods Hollow Road, Fitchburg, WI 53711 USA; 2 PerkinElmer Inc., Life Sciences and Technology, 940 Winter Street, Waltham, MA 02451 USA

**Keywords:** Spheroid, Organoid, Monolayer, Physiologically relevant, Assay, 3D, Imaging, HCS

## Abstract

Along with the increased use of more physiologically relevant three-dimensional cell culture models comes the responsibility of researchers to validate new assay methods that measure events in structures that are physically larger and more complex compared to monolayers of cells. It should not be assumed that assays designed using monolayers of cells will work for cells cultured as larger three-dimensional masses. The size and barriers for penetration of molecules through the layers of cells result in a different microenvironment for the cells in the outer layer compared to the center of three-dimensional structures. Diffusion rates for nutrients and oxygen may limit metabolic activity which is often measured as a marker for cell viability. For assays that lyse cells, the penetration of reagents to achieve uniform cell lysis must be considered. For live cell fluorescent imaging assays, the diffusion of fluorescent probes and penetration of photons of light for probe excitation and fluorescent emission must be considered. This review will provide an overview of factors to consider when implementing assays to interrogate three dimensional cell culture models.

## Introduction

**Figure 1.**
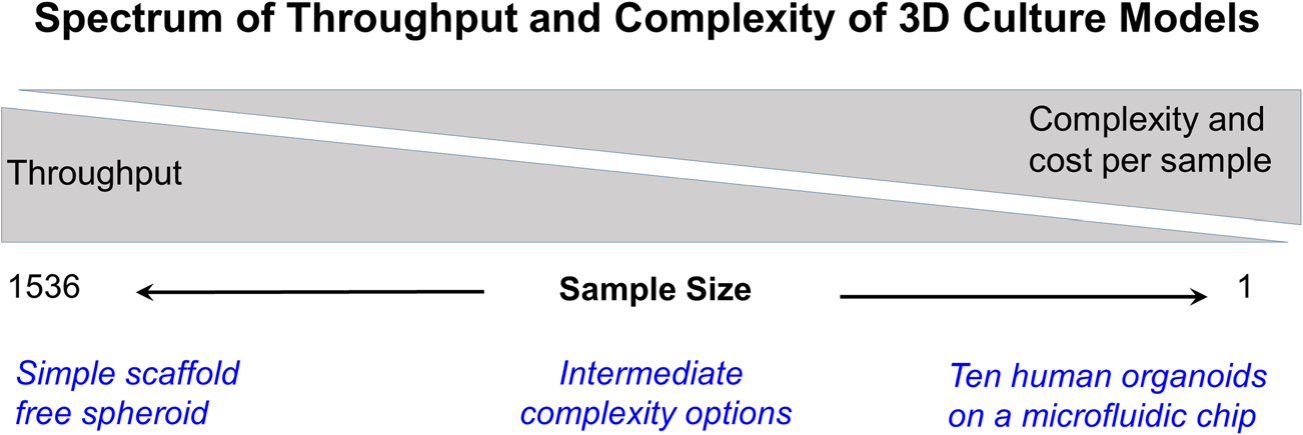
Balance of throughput with cost per sample and complexity of 3D culture models.

There is increased awareness that cells cultured in vitro as three-dimensional (3D) structures represent a more physiologically relevant environment compared to cells grown as a monolayer on a plastic surface. As a result of numerous published reports (Rimann and Graf-Hausner 2012; Edmondson *et al*. 2014; Fu *et al*. 2017) demonstrating that 3D culture models more accurately represent the in vivo situation, there has been a rapid adoption of a broad spectrum of simple to complex 3D models to study biological events. One of the challenges scientists face is the choice of a fit-for-purpose model that is as simple as possible, but as complex as needed to answer the scientific question being asked. Choice of a fit-for-purpose 3D culture model system often includes a decision between cost or throughput and complexity of the model system. Figure 1 is meant to illustrate that there is a spectrum of 3D culture models ranging from scaffold-free spheroids of cancer cell lines to complex microfluidic devices containing several organoids representing a “human-on-a-chip”.

At one end of the spectrum, simple spherical aggregates of cells (spheroids) can be formed in a scaffold-free environment by culturing cells in plates with non-adhesive surfaces (Madoux *et al*. 2017; Hou *et al*. 2018; Quereda *et al*. 2018) or by using a hanging drop technique (Messner *et al*. 2013). Cells cultured using those simple approaches often are forced to self-organize into spheroid shaped structures and eventually deposit a scaffold of extracellular matrix similar to the in vivo environment that supports a differentiated phenotype. Screening large numbers of samples using robotic liquid handlers to dispense a tumor cell line directly to 1536-well plates to form spheroids followed by incubation, addition of detection reagent, and recording data represent a high throughput, low complexity, low cost per sample option (Madoux *et al*. 2017). With that simplistic approach, the most widely used 3D culture model is to determine if a treatment influences the health or biology of the cells in the 3D model using a cell proliferation or viability assay (Comley 2017).

On the more complex end of the spectrum of models is the human-on-a-chip concept consisting of several microtissues or organoids. An organoid is being defined as “a collection of organ-specific cell types that develops from stem cells or organ progenitors, self-organizes through cell sorting and spatially restricted lineage commitment in a manner similar to in vivo, and exhibits the following properties: it has multiple organ-specific cell types; it is capable of recapitulating some specific function of the organ (e.g. contraction, neural activity, endocrine secretion, filtration, excretion); its cells are grouped together and spatially organized, similar to an organ” (Lancaster and Knoblich 2014).

A further level of complexity is added by including fluid flow to mimic the circulatory system connecting different microtissues in a logical order to enable metabolic interaction. Although that approach takes into consideration many factors and may more closely represent the in vivo situation, the tradeoff is the expensive and time-consuming processes required to generate organoids and support microfluidics and environmental control for a sample size of one.

Physically stretching culture models by incorporating movement to mimic the expansion and contraction of lung tissue (Huh *et al*. 2010) and the addition of immune cells to model an inflammatory response by lymphocyte activation and chemotaxis (Polini *et al*. 2019) are further examples of complexities that can be included in the 3D culture model system.

One approach that some consider to be a complex 3D culture model is the use of whole living organisms such as developing zebrafish embryos. In some respects, they have similar properties used in the human-on-a-chip model and have some of the same challenges when validating the performance of assays applied to whole fish. How closely the model represents human biology may be questioned; but the in vivo models do have their own internal microfluidics system with intact biological processes that provide the opportunity to study development and interaction of organ systems in their natural environment.

Most researchers employing 3D cell cultures are using models somewhere in between the two extremes of the spectrum of complexity. Choosing the most appropriate fit-for-purpose model involves compromise between the complexity and the cost or throughput. Researchers must consider whether the model has adequate complexity to answer the experimental question and whether the data from the experiment is worth the investment. Spheroids generated from a tumor cell line (e.g., HeLa) may lack important features contributed by fibroblasts or the nutrient environment in vivo. However, if the scientific question is to determine whether a treatment is directly cytotoxic, spheroids formed from a tumor cell line in a scaffold-free environment may be adequate for that purpose. If a test compound requires a metabolic conversion by one cell type to be detected as toxic to a second cell type, a slightly more complex multi-cell model may be required to detect the biological activity being sought. Similarly, if the activity of a test compound requires a contribution from the biology of the matrix components from fibroblasts or stromal cells, an intermediate complexity model incorporating more than one cell type is required.

The use of primary cells derived from patient biopsies to form spheroids reflects a greater physiological relevance compared to using a continuous cell line (e.g., HeLa, HCT116, or A549) that has been passaged for years. Pieces of tumor can be enzymatically dissociated into a single-cell suspension; then the cells processed to create uniform spheroids composed of multiple tumor and normal cell types (Lee *et al*. 2018; Phan *et al*. 2019). Alternatively, tumor samples can be sliced thin enough to allow sufficient nutrient supply, but thick enough to maintain the natural tumor architecture and used directly for ex vivo testing of drugs (van den Brand *et al*. 2017). Those methods have been used to create a personalized medicine approach to screen drugs to identify individualized therapeutic options for treating cancer. Although those methods have the added complexity of using a mixture of primary cells directly from the animal, they suffer from a limitation of the number of screening assays that can be done without the ability to expand the number of cells. Cell number expansion strategies such as growing xenographs in immune compromised mice have been used, but uniform expansion of all accessory cell types from the native tumor is lacking.

Recent advances have been made in optimizing the culture methods for using stem cells from normal and diseased patients to generate an unlimited supply of organoids containing multiple differentiated cell types closely reflecting in vivo biology. Researchers now can produce advanced 3D organoid models representing many human organs that are suitable for studying a variety of diseases in vitro (Aboulkheyr Es *et al*. 2018; Artegiani and Clevers 2018; Eglen and Reisine 2019). These techniques overcome many of the disadvantages of using primary cells derived from patient biopsies, most notably heterogeneity of samples and the limited availability of source material. The key issue to consider when using these stem cell approaches is the size, stage of development, and heterogeneity of the sample being tested.

## Characteristics of the sample

The broad spectrum of different kinds of 3D culture models creates a challenge when designing and selecting an assay that will accurately measure the desired parameter. The characteristics of the sample vary widely across the spectrum of 3D culture models. As the demand increases for assays to interrogate 3D culture models, there is an unmet need for assays verified to function with those models.

Among the most important factors to consider when verifying assay performance are the cell type, size of the 3D structure, and whether a scaffold material is part of the model. Not all cells in an individual 3D culture model are the same. When cells with the capacity to proliferate are used to form spheroids, the cells on the surface are undergoing growth and proliferation, whereas cells in the center of the spheroid may be quiescent or necrotic because they are starved of nutrients and oxygen due to limitations in diffusion of molecules that are constantly being used by the viable cells.

## Cell type

The inherent characteristics of each cell type cultured in a 3D environment must be considered. Different cell types have different growth characteristics that produce a variety of 3D structures. Some cell types cultured in a scaffold-free environment will form uniform spherical structures, while other cell types may form loose aggregates or hollow cysts upon differentiation. Cells that form tightly packed masses and secrete a dense extracellular matrix will likely be more resistant to uniform cell lysis compared to cells that form loose aggregates or hollow cyst-like structures. Tumor cells in 3D models generally continue to proliferate and form large 3D structures during extended incubation time, whereas primary cells without the ability to proliferate will form structures that are relatively stable over time. If an assay has been validated to work well with 350-μm diameter structures from one cell type, you cannot assume that it will work the same way with a different cell type. Each cell type and assay combination must be validated using appropriate controls to confirm that the assay is performing as expected and to minimize artifacts.

## Size

General assay categories to interrogate the biology in 3D culture models can be divided between those that utilize a specific detection chemistry commonly measured using a plate reader and those assays using imaging with a microscope. For both detection categories, the effect of the size of the 3D mass of cells and nature of the sample are critical parameters that must be considered when validating assays applied to 3D culture models. Whether the assay reagents or light signal will be able to penetrate through the mass of cells will affect the performance of the assay.

**Figure 2.**
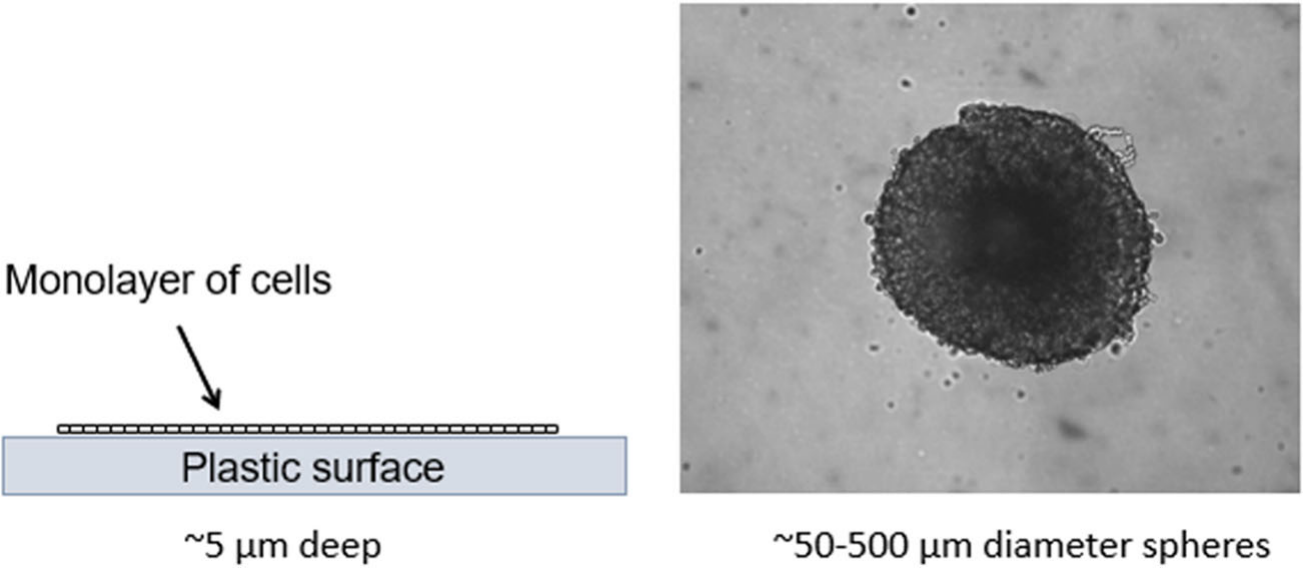
Hypothetical illustration showing a monolayer of cells on a plastic surface and an individual spheroid of cells. An assay reagent or small molecule probe has direct access to all cells in the monolayer, whereas the reagent must penetrate through multiple layers of cells (up to hundreds of micrometers) to reach the center of a spheroid.

Figure 2 illustrates that a comparison of the relative distance reagents must penetrate for a monolayer of cells versus a simple spheroid in a scaffold-free culture.

In a scaffold-free culture system, a monolayer of eukaryotic cells on plastic culture surface is ~ 5 μm deep, and assay reagents generally have direct access to the surface of all the cells in the monolayer. However, for solid spheroids, the reagents must be able to penetrate a distance equal to the radius of the sphere to reach all the cells. For example, for a spheroid with a 300-μm diameter, the reagents must penetrate through 150 μm of cell layers to reach the center. Among the spectrum of different 3D culture model systems being used, individual 3D structures may vary in size from less than 50 μm to several hundred micrometers in diameter (Li *et al*. 2016).

Release of markers from the center of 3D structures into the culture medium also must be considered. Lactate dehydrogenase (LDH) is a ubiquitous enzyme used as a marker of cell death. Upon cell death, LDH is released from the cytoplasm into the culture medium where it can be detected using plate reader assays. The movement of released LDH through the layers of cells to reach the culture medium on the outside of 3D structures should be confirmed. An orthogonal approach demonstrating the correlation of penetration of a small molecule fluorescent vital dye probe is an orthogonal method that can be helpful to verify efficient release of LDH.

A dilemma for researchers beginning to use 3D culture models is that most commercially available assay reagents were developed using cells in suspension or cultured as a monolayer. The reagent design and formulation optimization did not consider large 3D structures. As researchers switch to using more physiologically relevant models, it is up to the individual investigators to validate the performance of assay reagents applied to 3D culture models.

**Figure 3.**
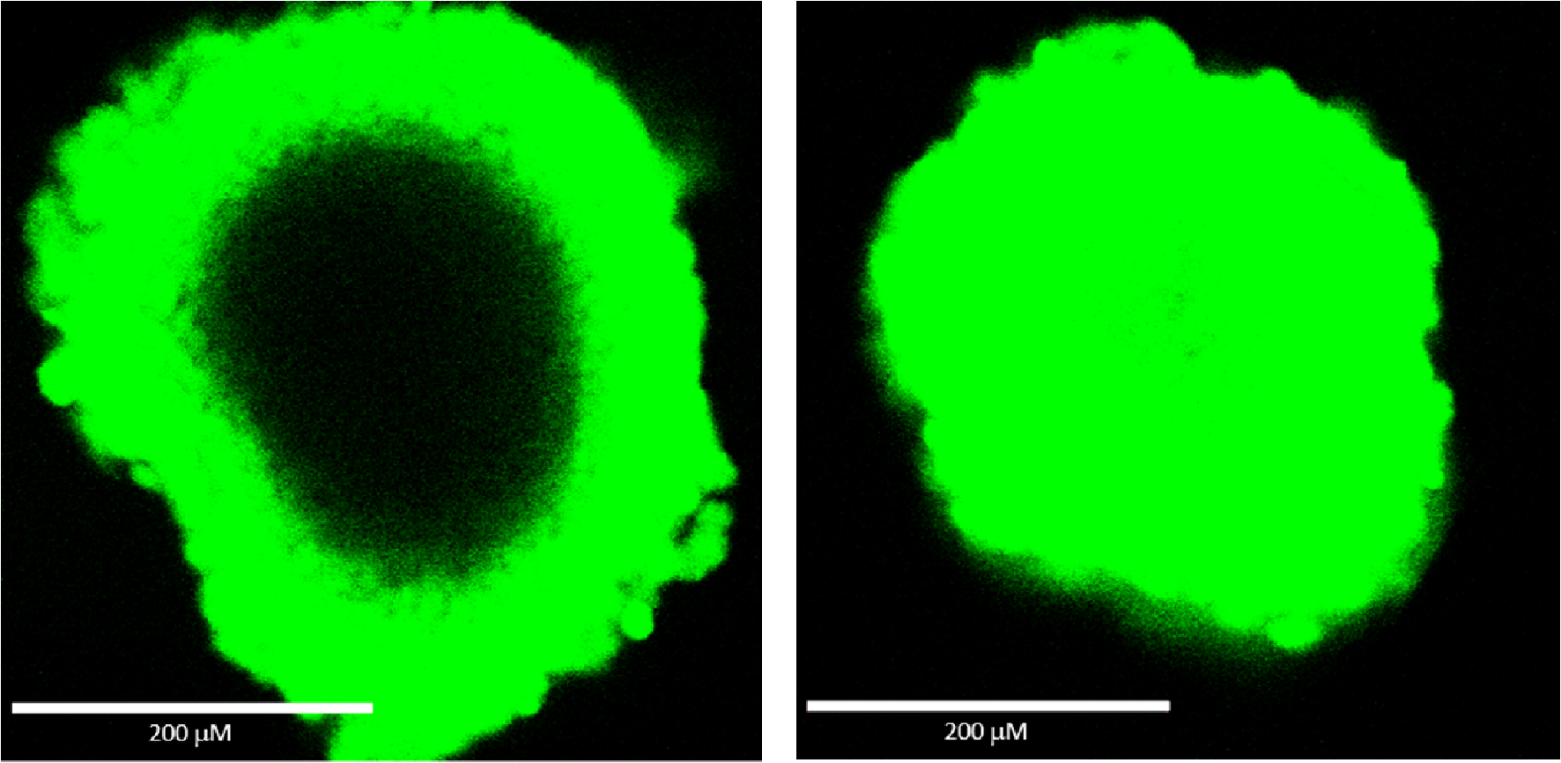
Comparison of the ability of two different ATP assay reagents to lyse cells in ~ 350-μm diameter spheroids measured using staining with a vital fluorescent DNA binding dye. HCT116 cells were added to a hanging drop device (GravityPLUS™ 3D cell culture system from InSphero) and cultured for 4 d to produce ~ 350-μm diameter spheroids. The ATP assay reagents and nonpermeable DNA dye were combined and added according to the manufacturer’s recommended methods. The samples were shaken for 5 min followed by 25 min incubation before laser confocal microscopy was used to record photographs of the two spheroids using the same conditions.

Figure 3 shows the results of an experiment comparing the ability of two different ATP assay reagents to lyse cells in spheroids formed from a human colon tumor cell line (HCT116) in a scaffold-free environment. For this experiment, a fluorogenic DNA binding dye (which only penetrates dead cells that have lost membrane integrity) was combined with the detergent-containing ATP assay reagents. If the detergent containing reagent lyses the cells, they should stain green by the vital dye.

The dark (unstained) center of the spheroid shown on the left suggests incomplete cell lysis, whereas the uniform green staining of the spheroid on the right suggests more efficient lysis of cells in the center of that spheroid. This example illustrates the need to verify assay reagent performance.

There are limits to the ability to extract ATP from samples using homogeneous methods that were designed for HTS screening of monolayers of cells in microwell plates. Those limits can be demonstrated by comparison to more efficient nucleotide extraction methods. Acid treatment is one of the methods that has been used for decades for extracting nucleotides from tissue samples. Tissue homogenization at low pH will precipitate proteins, inactivate ATPases, and provide an environment to stabilize the nucleotides for subsequent quantitation by HPLC or luciferase assay after pH neutralization. Acid extraction techniques are considered the gold standard for recovery of ATP from samples and as such can be used for comparison to determine the percent recovery of ATP by other methods.

Comparison of trichloroacetic acid and a detergent-containing assay reagent (designed for HTS of low numbers of cells) applied to a series of spheroids of increasing diameters demonstrated similar recovery of ATP from spheroids up to ~ 350 μm; but there was a trend of lower percent (less efficient) recovery of ATP from spheroids with a diameter above ~ 350 μm (data not shown). That observation suggested there are limitations in the ability to lyse cells and recover ATP from large spheroids using the standard luminescent ATP assay procedures designed for use with a monolayer of cells.

The results of those experiments (and other work attempting to validate off-the-shelf commercial assays) led to efforts to generate improved reagents designed for use on 3D culture models. A general set of recommendations for an approach to adapt off-the-shelf assays for use with more difficult to lyse 3D structures includes the following: optimizing the reagent formulation to increase the ability to lyse cells, incorporating more vigorous physical disruption of the sample after the reagent has been added, and increasing the incubation time in the presence of the detergent-containing reagent.

Improvements made for the ATP assay incorporated all three of those approaches. The reagent was reformulated to modify the detergent ingredients to more efficiently lyse cells and extract ATP from large 3D structures, the assay protocol was modified to include a 5 min mixing step using an orbital shaker, and the incubation period was extended to lengthen the time of exposure of the sample to the detergent-containing reagent. The approach to increase the lytic capacity of the ATP detection reagent with strong detergents required using a stable form of recombinant firefly luciferase that can withstand detergent, heat, and pH shifts (Hall *et al*. 1998).

Although that combination of approaches represents a good first step for improving assays applied to 3D culture models, it may not work for all reagents. There are cases where reformulating the reagent to change the detergent concentrations is not compatible with the stability of the marker being measured. For example, caspase-3 enzymatic activity (which is commonly used as a marker of apoptosis) is known to be sensitive to high concentrations of detergent. In that case, the logical approach is to increase the physical disruption and length of incubation with the same lytic reagent commonly used for monolayer culture models (Wilson *et al*. 2019).

The effectiveness of cell lysis can be monitored using the same fluorescent vital dye approach as described for the ATP assay. Combining a fluorescent vital dye with the luminescent caspase detection reagent and using several minutes of vigorous physical disruption with an orbital plate shaker demonstrated improved cell lysis similar to images shown in the right panel of Fig. 3 for the ATP assay. Morphological observation of green staining of cells that have lost membrane integrity can be used as additional evidence the lytic assay reagent is effectively lysing cells.

## Orthogonal methods

Do not rely on a single approach for determining assay performance with 3D culture models. Confirming results using an orthogonal method is a good strategy to identify artifacts and avoid misinterpretation of results. Applying more than one method to measure the same marker can serve as a control and confirm results. For example, comparing the orthogonal methods of acid and detergent extraction of ATP uncovered a performance limitation in the luminescent assay that was originally designed for HTS of monolayers.

Another approach is to measure a different marker of cell viability rather than use a different method to measure the same marker, for example, comparing ATP content and MTT tetrazolium reduction assays. Both assays have been used extensively to measure cell viability; but each method has drawbacks which are often overlooked. MTT can be cytotoxic (Riss *et al*. 2016) and cannot be used for individual spheroids because of limits in detection sensitivity. The luminescent ATP assay may have interference by light quenching compounds or luciferase inhibitors (Auld and Inglese 2016).

**Figure 4.**
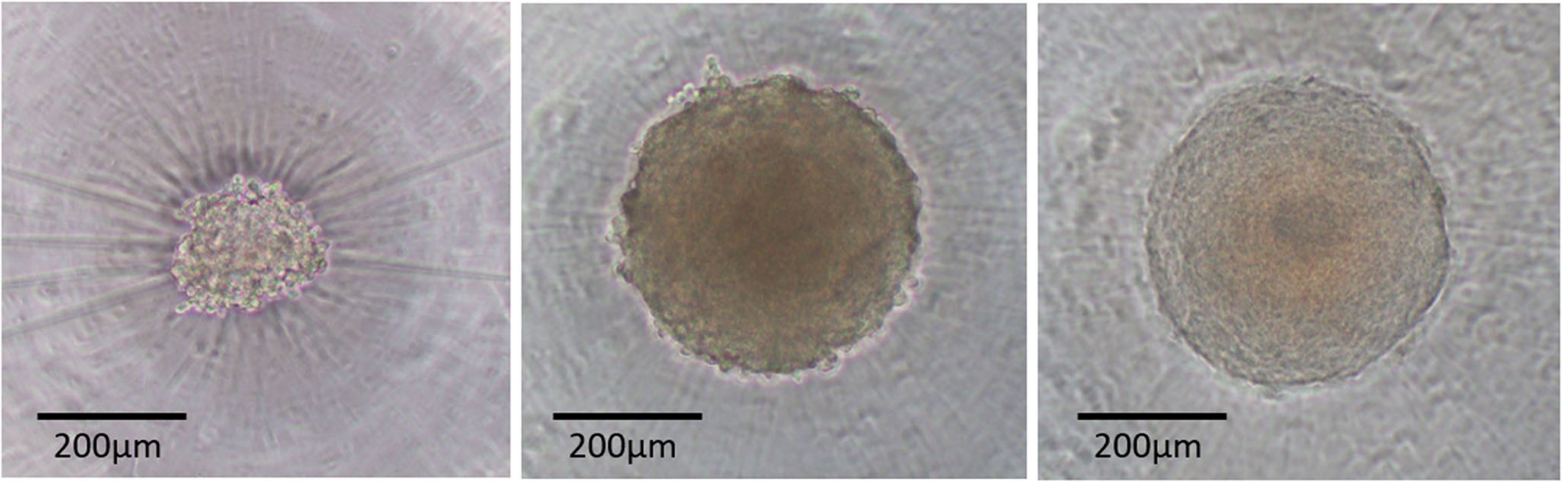
HCT116 cells in 100-μL medium supplemented with 10% FBS were added to corning spheroid plates (Cat# 4520; 96-well black opaque wall with a clear ultralow attachment rounded bottom). After settling, the wells were photographed using phase contrast microscopy (*left image*) and cultured in a humid environment at 37°C, 5% CO_2_ for 4 d to allow spheroid formation. The same wells were photographed again (*center image* approximately 330 μm); then 100 μL of the re-formulated version of the ATP detection reagent (CellTiter-Glo® 3D cell viability assay cat. #G9681) was added to each well, and the plate was shaken using an orbital mixer at 600 rpm for 10 min. The samples were photographed again after mixing (*right image*).

The above example of using imagining of lysed cells also is a valuable orthogonal approach for confirming results. However, low magnification viewing of spheroids with phase contrast microscopy also can be misleading. The observation of a spherical object remaining in the sample well following treatment with a lytic reagent may lead to an interpretation that the assay did not work. Figure 4 shows the same sample well photographed at different times.

The image on the left was recorded the day the cells were added to sample well having an ultralow attachment surface. The center image was recorded after 4 d of incubation to allow spheroid formation. On day 4, detergent-containing ATP detection reagent was added, and the plate was shaken for 10 min on an orbital shaker to thoroughly mix contents and include some physical disruption. The image on the right was recorded after reagent addition and mixing. The outline of a spheroid structure can clearly be seen in the image on the right; however, experiments using the same cell line to compare acid extraction with the detergent-containing luminescent detection reagent suggested that essentially all the ATP has been extracted from spheroids of that size range using the detergent-containing ATP detection reagent. These data (as well as the images from Fig. 3) suggest the plasma membranes of the individual cells within the spheroid have been lysed to release ATP although the gross structure of the spheroid remains relatively intact. The cytoskeletal structure and basic elements of the extracellular matrix may remain relatively intact even if individual cell membranes have been lysed. In situations when the results of two orthogonal assays do not agree, it is advisable to confirm results using additional methods.

## Sample mass

The total mass of cells or biomatter in the sample also must be considered when choosing assays for 3D culture models. The total number of cells in 3D culture models can vary widely, ranging from hundreds of cells in individual spheroids to millions of cells in large reconstructed models used to mimic skin.

Assays to interrogate an individual spheroid require greater detection sensitivity because of the small number of cells. For example, an ~ 200-μm diameter spheroid might contain 1500–2500 cells, whereas a confluent monolayer in the bottom of a single well of a 96-well plate might contain over 10,000 cells. The assay must be able to detect a significant change in the marker being measured in a small population of cells. Adequate detection sensitivity generally can be achieved by microscopic imaging individual cells containing fluorescent markers or by using fluorescent or luminescent assay endpoints detected using an appropriate plate reader. However, colorimetric absorbance assays using tetrazolium reagents such as MTT typically do not have adequate detection sensitivity to be useful to monitor viability changes in individual spheroids containing only ~ 1500 cells.

Combining numerous spheroids harvested from a mass production step and dispensing into an assay plate can overcome the sensitivity issue with the MTT assay; but alternate approaches using fluorescent or luminescent plate reader compatible assays have adequate detection sensitivity to record data from individual spheroids or organoids.

The total mass is also important to consider when the sample is large. The quantity or concentration of the marker to be measured may be beyond the linear range for an assay reagent detection chemistry designed for monolayers of cells. Although there are examples of using several commercially available off-the-shelf assays being used on large dermal constructs (Idrees *et al*. 2018), detailed description of the validation for non-intended use is often lacking. For example, some dermal equivalent models that are assembled on filter inserts and used for skin irritation or cosmetics testing may approach ~ 100-μm thick and ~ 1 cm diameter. That represents a far greater total mass and a different set of challenges compared to individual spheroids or a monolayer of cells in a 96-well plate.

The recommended method of performing a viability assay on these dermal equivalent models is the MTT tetrazolium assay (OECD 2019). The assumption is that the small molecule probe (MTT) uniformly penetrates the layers to enter viable cells where it becomes biochemically reduced to form an intensely colored formazan precipitate. The formazan product is subsequently extracted using acidified isopropanol and the 570-nm absorbance recorded using a plate reading spectrophotometer. Uniform penetration of MTT and uniform extraction of the formazan are parameters that could be of concern. Some dermal models contain a gradient of differentiated squamous epithelial cells representing in vivo keratinization with a cornified layer that does not maintain the capacity to reduce the MTT into formazan. Although this model accurately represents the in vivo situation, the heterogeneity from one side to the other and the relatively large mass of the sample are characteristics that must be considered when selecting an assay and interpreting data. Histochemical sections can be used to identify the distribution of the formazan precipitate and demonstrate penetration of the MTT probe; however, it would be tedious to section and analyze a large number of samples.

In general, small molecule cell permeable probes such as MTT or calcein-AM to stain live cells or membrane impermeable vital DNA dyes to stain dead cells can eventually penetrate (diffuse) into the center of 3D culture models; but it may take hours (Sirenko *et al*. 2015). Adequate incubation time to ensure uniform penetration should be verified to avoid misinterpretation of results. Sparse staining in the center of a spheroid may be the result of fewer viable cells, lower metabolic activity of viable cells present, limited diffusion of the probe to the center of the mass, or limitations in detection of the optical signal which could result from limited penetration of light to excite the fluorophore or scattering of the emitted fluorescent signal. One approach to investigate probe permeability is to multiplex with an orthogonal method such as using a vital DNA binding dye. A decrease in fluorescent signal from a viability probe such as calcein-AM should coincide with an increase in signal from a vital dye probe designed to detect dead cells. Much larger probes such as antibodies and antibody fragments also have been reported to penetrate 3D cell culture models and tumor tissue in a time and concentration-dependent manner (Thurber and Wittrup 2008); however, regardless of the procedure used, each probe should be independently validated to ensure the expected distribution and assay performance.

## Scaffold-free models

Many 3D culture techniques can create simple spheroids using a scaffold-free approach. The predominant techniques to create uniform-sized spheroids include using ultralow cell binding surfaces in individual U-bottom wells, the hanging drop method, and applying a gyratory shaking motion of cultures for weeks allowing aggregation of cells and differentiation into neurospheres (Pamies *et al*. 2017). There are also flasks or assay plates with arrays of hundreds of ~ 500-μm diameter microcavities that support formation of an individual spheroid per cavity (e.g., Elplasia® plates from Corning).

Other scaffold free methods employ various surface modification techniques to create micropatterns of subcellular sized squares, honeycombs, or other shapes on polymeric films that support spheroid formation by altering adhesion of cells to the modified surface (e.g., NanoCulture® Plate from MBL International Corp.).

T-flasks with a large ultralow binding surface are available to generate millions of spheroids. However, the latter two scaffold-free methods are examples that tend to create heterogeneous clusters of cells that have a broad size range. A wide heterogeneity in size of the structures may lead to unacceptable differences in cell responsiveness to treatments among the population of 3D structures.

It is possible to select an acceptable range of sizes using sieving techniques (Shi *et al*. 2018). However, the procedural complexity of selecting a population of uniformly sized spheroids and reproducibly dispensing them into assay wells are factors to consider when choosing a fit-for-purpose 3D culture model. Researchers can make a choice whether it is more acceptable to generate an individual spheroid per well and perform the assay in the same plate or to mass produce spheroids in T-flasks and subsequently attempt to harvest a uniform size range and dispense a uniform number per well into assay plates.

## Scaffolds

Many 3D culture models incorporate the addition of some sort of scaffold material to mimic the in vivo architecture supporting cells. Including a scaffold material in the cell culture model may be required to improve the physiological relevance and achieve the desired biological responsiveness of the model; but it also adds to the complexity of both culturing and assaying the sample. There is a wide range of approaches to provide a scaffold, and many different types of material are used ranging from chemically defined inert structures (Nguyen *et al*. 2017) to decellularized organs (Tapias and Ott 2014; Germanguz *et al*. 2019). Inert scaffolds simply provide a physical structure for cells to attach to or grow into, and some are designed to biochemically mimic the native extracellular matrix environment of the tissue model being built. In many cases, the scaffold can be a barrier to penetration of reagents, release of biomarkers into the culture medium, penetration of light, or positioning of cells outside of the working distance of an objective lens. Regardless of the composition, the barrier properties of the scaffold material must be considered when validating assay performance.

For example, there are inert structural scaffolds such as a 200-μm thick porous polystyrene (e.g., Alvetex® Scaffold from AMSBIO, Cambridge, MA) that provides a porous structure for cells to grow into; however, it is not compatible with microscopic imaging unless the sample is removed and sectioned using histochemical techniques. In contrast, the use of electrospinning to deposit nano- and micro-fibers creating a translucent ~ 50-μm coating of surfaces can serve as an inert scaffold that is compatible with imaging techniques (Mimetex from AMSBIO). Although inert scaffolds are chemically defined and can be fit-for-purpose, they lack the direct interaction of cells with native extracellular matrix (ECM) components unless those components are included in some form in the culture medium or they are deposited by the cells over time.

Scaffold materials that mimic the environment of the native extracellular matrix (ECM) may be more complex, but they are also more physiologically relevant than inert scaffolds. Native scaffold materials are commonly used in the form of a hydrogel which is composed of a network of polymer chains held together by crosslinking.

The use of collagens from animal sources was among the early and popular choices to provide a biological scaffold that exists as a hydrogel (Bissell *et al*. 2003; Simian and Bissell 2017). A widely used version developed at the NIH (Kleinman and Martin 1989) is a urea extract of the Engelbreth-Holm-Swarm (EHS) mouse sarcoma that expresses many extracellular basement matrix materials including laminin, collagen IV, nidogen, heparan sulfate proteoglycan, and entactin. That tumor extract which was named Matrigel has been demonstrated to support the formation and differentiation of a wide array of 3D cell structures. Matrigel is commonly used as a default source of basement membrane components. It is used in a manner similar to that of fetal bovine serum to supplement culture medium. That is, it works like magic to produce a desired effect despite being an undefined animal-derived material.

The US patent that described the EHS extract claims the addition of a protein extract in a concentration of at least 3.7 mg/ml, which brings up an important point. Most hydrogels are greater than 90% aqueous. Although the lattice structure of hydrogels will limit diffusion and slow down the penetration of small molecule probes, in general, assay reagents will eventually penetrate unless there is some chemical or non-specific binding to the probe.

Because Matrigel is a crude extract from tumors and its constituents are not entirely defined, it is subject to batch-to-batch variation in the type and amount of matrix proteins. It is also known to contain contaminating growth factors such as basic fibroblast growth factor, epidermal growth factor, insulin-like growth factor 1, transforming growth factor beta, platelet-derived growth factor, and nerve growth factor (Vukicevic *et al*. 1992; Hughes *et al*. 2010). Although reduced growth factor preparations of the EHS extract are commercially available, there is still variability due to many other unknown proteins and components including nucleic acids (Talbot and Caperna 2015). The usefulness of vital DNA binding dyes as a reagent to label dead cells is limited in Matrigel because of background fluorescence, likely due to DNA contamination of the animal-derived tumor extract.

It has been known for years that EHS extract may contain multiple growth factors (Vukicevic *et al*. 1992). Approaches to more clearly define the influence of scaffold materials and to provide alternatives to animal use have resulted in the generation of algae or plant-derived materials such as alginate, agarose, and nanofibrillar cellulose derived from Birch trees (Bhattacharya *et al*. 2012). Yet another approach to define extracellular matrix components is to use recombinant proteins (e.g., laminin fragments) or chemically defined synthetic materials including peptides and polymers of polyethylene glycol (PEG) modified to contain the Arg-Gly-Asp (RGD) amino acid sequence for binding to cell surface integrins.

There is a movement to use synthetic chemically defined polymers as scaffold components to avoid batch-to-batch variability and the unknown effects of the hundreds of proteins present in crude tumor extracts. Chemically defined synthetic biomaterials exist that can be optimized to maintain cellular health and support differentiation in 3D cultures. Polyethylene glycol (PEG) monomers are cross-linked with bioactive cell adhesion peptides to form hydrogels that can control properties of mechanical stiffness, cell adhesion, and degradability (Nguyen *et al*. 2017). The use of chemically defined components will simplify both the culture model and the assays used for interrogation of 3D culture models. Properties such as sheer thinning and the ability to breakdown the scaffold using defined procedures for recovery of cell structures will lead to more reproducible assay results.

## Considering limitations of models that incorporate microfluidics

Engineered devices that provide a flow of culture medium in 3D models add another level of complexity. There are a variety of microfluidic chip devices that connect the culture medium among organoid chambers to mimic vascularization and represent the in vivo environment. Most assays used to interrogate live samples in microfluidic devices in real time rely on imaging with fluorescent probes; however, there is also an opportunity to use plate reader assays to measure markers released from cells by sampling small aliquots of the culture medium over time. The major challenges that must be considered include reproducibly removing and handling microliter size samples for analysis and the availability of an assay with adequate sensitivity to detect the desired marker in microliter size samples.

There may also be a concern about the availability of the biomarker in the sample of culture medium. It may take time following necrosis for large cytotoxicity markers such as lactate dehydrogenase (LDH) to reach a steady-state concentration in the culture medium. Applying an orthogonal approach such as using a small molecule fluorescent vital dye can be used to compare the relative kinetics of release of LDH vs. the appearance of stained (dead) cells.

### Optical microscopy and automated solutions to interrogate 3D cell models

**Figure 5.**
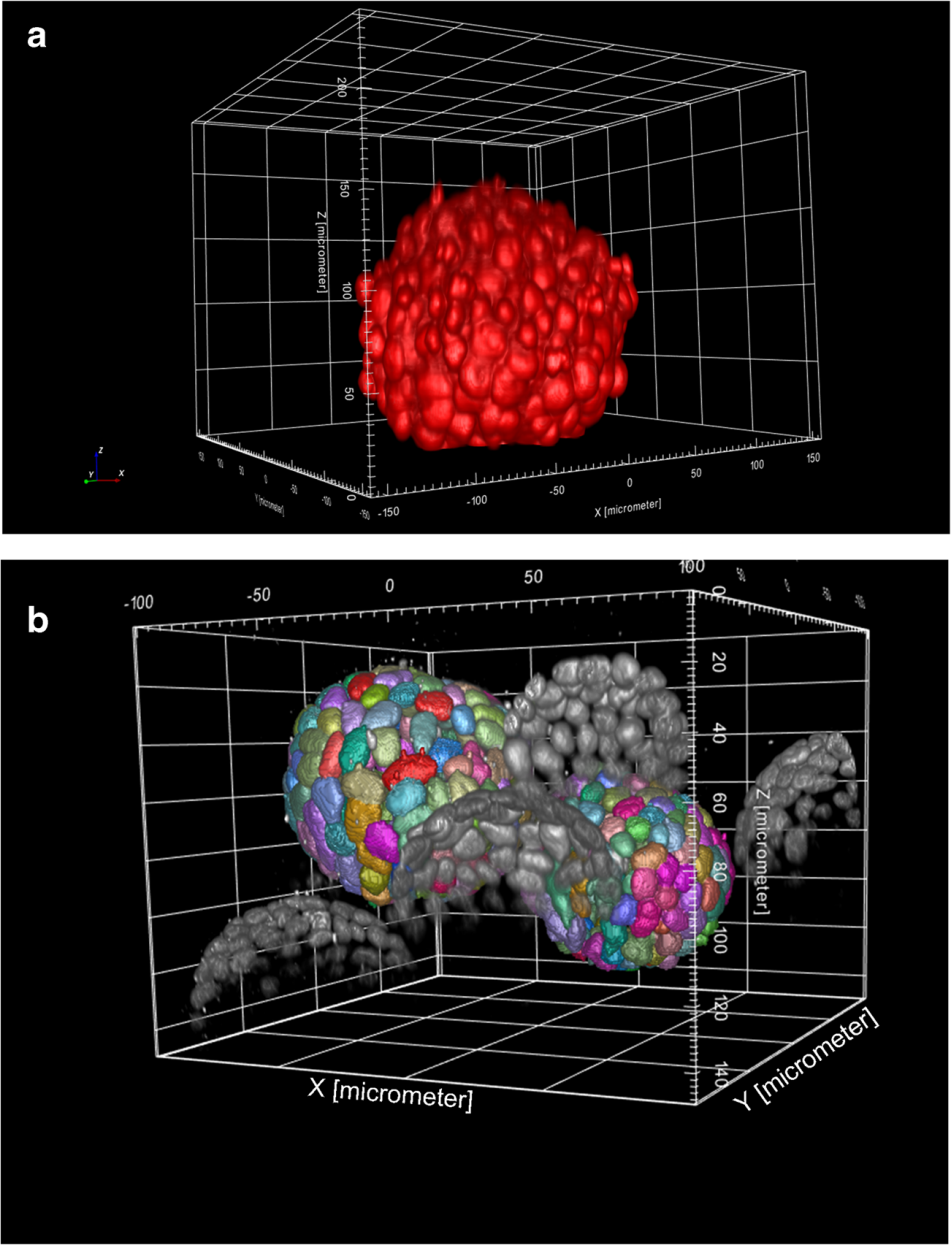
(*Top*) 3D reconstruction of a Chinese hamster ovary (CHO) spheroid labeled with DRAQ5™ and pseudocolored red. Image stack was captured with × 20 water lens on Opera Phenix (PerkinElmer) HCS imager; 0.8-μm z-space intervals, 301 slices. (*Bottom*) 3D reconstruction of Madin-Darby Canine Kidney (MDCK) cysts. Nuclei labeled with DRAQ5. Image analysis overlay (rainbow colors) of intact cyst not touching the boundaries. Image stack was captured with × 20 water lens on Opera Phenix HCS imager; 0.8-m z-space intervals, 301 z-slices.

The recent expansion of using in vitro 3D cell model systems in part is being exploited by the outcomes of new microscopy imaging techniques to better understand the mechanisms or mode of actions (MoA) following cellular perturbations. The technologically advancements in microscopy instruments and computational power to interrogate 3D cell models have improved quantification and detection deeper into tissue with greater sensitivity and have simultaneously reduced the time to capture images in some instances, e.g., high content screening (HCS) (see Fig. 5). However, there remain several challenges as highlighted in a commentary by key opinion leaders who discussed the strengths, weaknesses, opportunities, and threats (SWOT) analysis in HCS 3D imaging (Carragher *et al*. 2018). The authors pointed out the need for collaborative spirit between academia and industry to promote guidelines for imaging 3D cellular models, education, communication, and transparence of the value of new 3D cell model systems.

**Figure 6.**
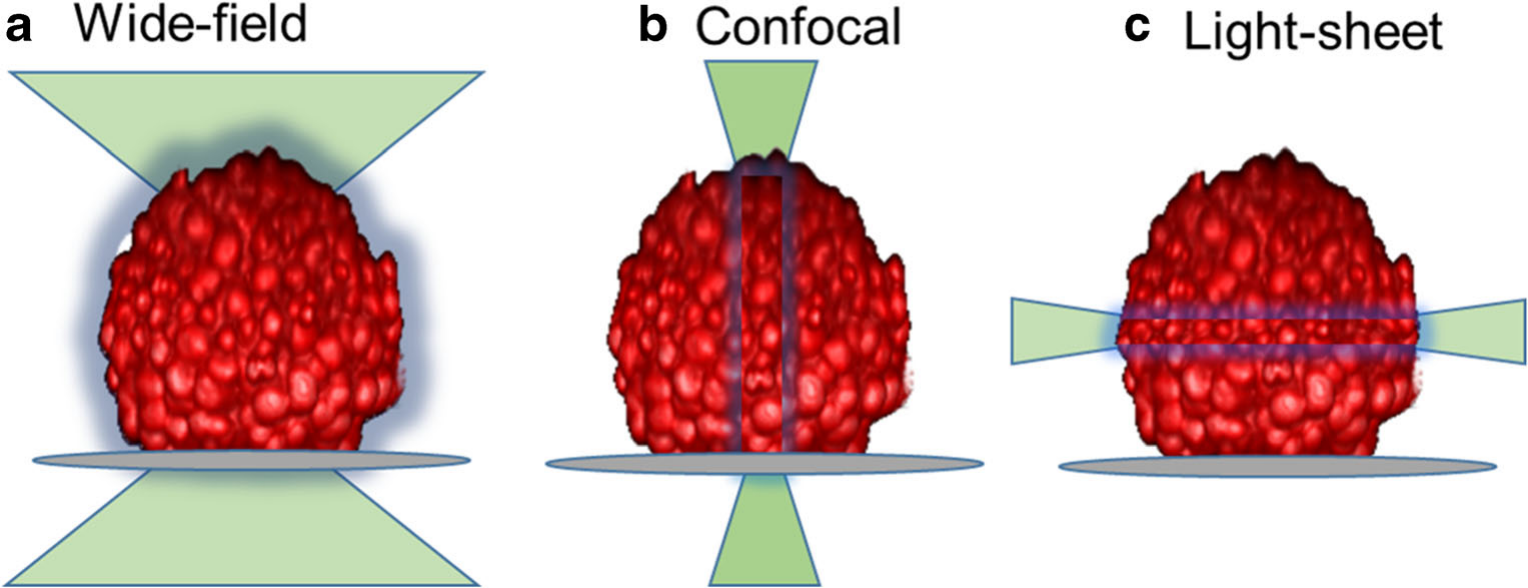
Illumination from different microscopes. (*a*) Broad illumination from wide-field microscope; (*b*) pinpoint vertical illumination from confocal microscope; (*c*) narrow horizontal illumination from light-sheet microscope.

**Figure 7.**
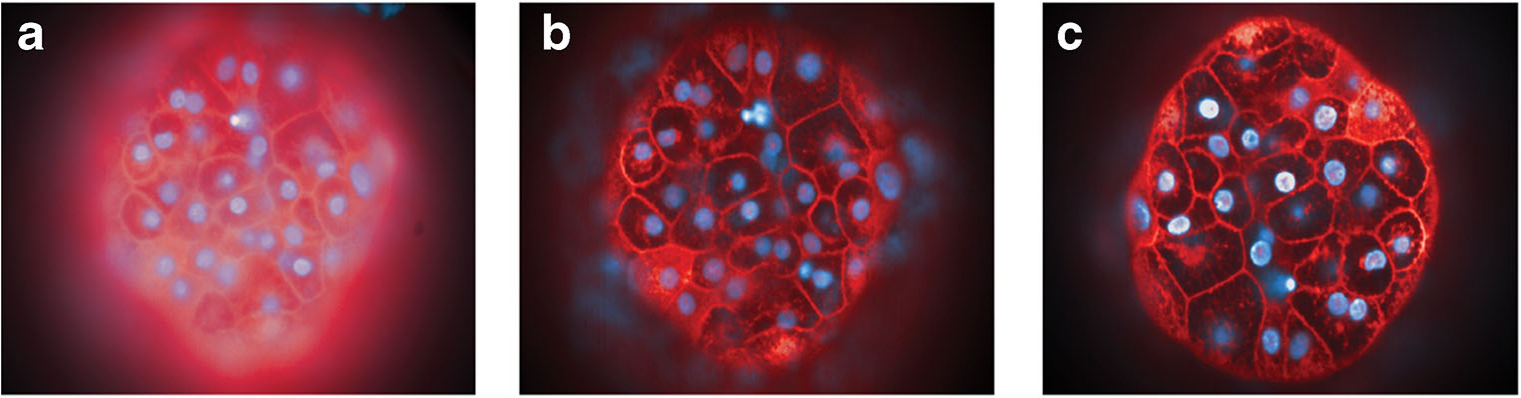
Maximum intensity projection reconstruction of 3D images of InSphero 3D InSight™ human liver microtissues. Images captured and rendered on Operetta CLS and Harmony software (PerkinElmer). Cells labeled with Hoechst 33342 (*blue*) and CellMask Deep Red (*red*). (*a*) × 40/0.75 NA wide field; (*b*) × 40/0.75 NA confocal; (*c*) × 40 water lens/1.1 NA confocal. Image courtesy of PerkinElmer ©2015–2020 PerkinElmer, Inc. All rights reserved. Printed with permission.

The three most common optical sectioning microscope systems used today for imaging 3D cell models include (1) confocal, (2) high resolution microscopy that includes light-sheet-based instruments, and (3) to a lesser degree wide-field croscopy (see Fig. 6). Other notable microscopy systems are scanning and transmission electronic microscopy (TEM). The TEM system can achieve 0.05-nm resolution; it does so only in very thin sections of approximately 100 nm and is very time-consuming to generate data, therefore not ideal for 3D volume analysis, whereas scanning electronic microscopy (SEM) can measure 3D surfaces in low throughput fashion. While magnetic resonance imaging (Lauterbur 1989) and radioactive computed tomography (Kalender 2006) provide contrast imaging for observing 3D structure in living tissue, the millimeter resolution and throughput are not ideal for higher-throughput basic and drug discovery research. A stereomicroscopy instrument relies on the observation of two independent optical paths and two intact functional “human eye” detectors to visualize 3D structures, but are unable to capture the same observed 3D images with an electronic camera for any post-image acquisition visualization and quantitation. High resolution microscope system that include but are not limited to structured illumination microscopy (SIM), stimulated emission depletion microscopy (STED), super-resolution photoactivated localization microscopy (PALM), and stochastic optical reconstruction microscopy (STORM) have excellent axial resolution ranging from approximately 10 to 100 nm (see Table 1). However, in most cases, they require significant sample preparation before imaging and/or are slow in generating image data for higher-throughput experiments. Mass spectrometry matrix-assisted laser desorption/ionization (MALDI) and secondary ion mass spectrometry (SIMS) technology otherwise referred to as 3D imaging mass spectrometry (IMS) advancements in recent years are remarkable with the capability of scanning large areas with multiple probes and axial resolution approaching 1 μm, but the technology still lacks speed and higher-throughput acquisition (Seeley and Caprioli 2012; Buchberger *et al*. 2018). Light-sheet fluorescent microscopy (LSFM), perhaps first described as an ultramicroscopy at the turn of the 1900 century (Siedentopf and Zsigmondy 1902), was refined with development of orthogonal-plane fluorescent optical section in 1993 (Voie *et al*. 1993), to its current name first described as LSFM in 2004 (Huisken *et al*. 2004). Since being introduced, light-sheet microscopy has undergone rapid expansion and grown in popularity over the last decade in academia with stunning 3D volumetric images with improved temporal and lateral resolution breaking the Abbe’s diffraction barrier limit of traditional light microscopy systems with axial resolution of roughly 250 nm (d = λ/2) (Abbe 1873). More recently, lattice light-sheet microscopy developed by Eric Betzig’s lab (Chen *et al*. 2014) along with their most recent advancement using adaptive optics to remove distortion (Liu *et al*. 2012) has improved sample preparation requirements making it more conducive to interrogate the native cellular environment reaching spatiotemporal resolution close to 20 ηm. Generating images with light-sheet microscopy modalities still requires appropriate sample preparation that is simpler than other high resolution microscopy methods; however, the image capturing process is still lengthy and not reduced to practice for automated high throughput of multiple samples that would revival HCS imaging. Confocal imaging systems remain the primary workhorse instrument for higher-throughput 3D microscopy, and they are found throughout most research laboratories making these the most popular microscope systems available to researchers for optical sectioning of 3D cell models. In most cases, the configuration of confocal microscope systems equipped with a 100x objective lens can theoretically achieve nearly 200 nm axial resolution. However, not all objective lenses are created equal and resolution is dependent on unique design with different magnification powers, NA, working distance, chromatic corrections, air or immersion solutions of water, oil, or glycerol with specification to work with thin microscope coverslips or may be equipped with a correction collar for fluctuations in sample surfaces such as thick-wall plastic surfaces found in some microplates. In most cases, low magnification objective lenses have lower NA, thus reducing amount of light entering the microscope and its overall potential resolution, while higher magnification lenses have NA approaching 1.0 or beyond with immersion fluids to enhance its capability. When the refractive index matches the biological media, this results in increase light throughput and reduced light scatter to the microscope optics resulting in improved resolution of the captured image (see Fig. 7). One of the limiting factors of confocal and wide-field microscope systems is the depth of focus penetration of light into the tissue sample that is directly dependent on the sample preparation and the optical configuration of the microscope system. In live cells, confocal imagers can capture bright fluorescent probes up to approximately 50- to 200-μm thick and slightly more when two-photon excitation light source is used, but again this is variable and directly dependent on sample preparation of the cells and probes, and the optical configuration including the NA of the objective lens in the system. In a process of fixing and clearing tissues or cells to reduce light scattering from interference with lipids, hemoglobin, myoglobin, or melanin in tissues, confocal microscopy can go well beyond 200 μm to millimeter depths with many of the traditional preparation methods, but these clearing approaches may alter cellular morphology (Richardson and Lichtman 2015).

**Table 1:** Generalize comparison of microscopy modalities for resolution, speed, and throughput

Microscope	Resolution	Speed and throughput
Wide field and confocal	250 nm	High
High resolution (PALM, STORM, SIM, STED)	10–100 nm	Slow
Light sheet	20 nm	Slow
SEM	0.4 nm	Very slow
EM	0.05 nm	Very slow

High content screening (HCS) imaging instruments were designed as an automated robotic high-throughput system equipped with wide-field or confocal optical capabilities of traditional microscopes for a non-biased walkaway approach for generating imaging data at an unprecedented rate of nearly a half million images in a day (Trask and Large 2001; Buchser *et al*. 2004). HCS systems are routinely used as workhorse instrument for chemical and drug screening centers throughout academia, government agencies, biotechnology, and pharmaceutical industries. The advantage of these systems is the unbiased approach of image acquisition process and statistical robustness of automated image analysis segmentation to measure hundreds or thousands of individual cellular objects in unprecedented time. This is unparalleled to traditional standalone microscopy modalities previously described for speed, sensitivity, and throughput to generate data from pixels. In screening labs, HCS imaging devices typically use ANSI/SBS standard microplate formats (Auld *et al*. 2020) that include 6-well plates to 1536-well microplates that are automatically delivered to the instrument with robotic arm devices for 24/7 operation. Many HCS imagers also are adaptive for microscope slide imaging (Li *et al*. 2020) and other specialized plate formats such as organ-on-a-chip devices. In most cases, imaging 3D cell models with HCS instruments use spinning disk confocal technology for rapid image capture to remove out of focus light while reducing photo destruction of the fluorescent probe and cell damage that is more typical in confocal point scanning devices. Due to the engineering and design of most HCS imaging systems, a disadvantage is not all cell model systems can be imaged if they do not follow the ANSI/SLAS microplate standard or other design restriction from the manufacture specifications of the HCS imaging instrument.

### Imaging 3D cell models

There are several imaging approaches and strategies to interrogate 3D cell models, none of which are standardized due to compounding factors of the vessel or microplate, cell model, probes, instrument optical sectioning properties, and associated software. Attaining optimal 3D image data is directly dependent on several factors with each step in the process of imaging 3D cell models requiring careful consideration that involves the experimental design to determine the vessel or microplate to seed cells, supporting environment, reagents for labeling, the instrument for detection, and image and data analysis to quantitatively measure biomarker probes to address biological questions. While each of these requirements is considered independent of one another, it is an aggregated approach with its overall success of an experiment contingent on each interaction. Considerations for imaging 3D structures starts with the cell model and supporting environment of the vessel or microplate in HCS imaging studies.

Vessels such as microscope slides, chamber slides, or petri dishes with coverslips are useful tools for basic research studies using standalone microscope systems but are not intended for higher-throughput experiments when experimental design and outcomes require multiple treatment conditions that are often used in screening. In this case, the use of microplates consisting of 6, 12, 24, 48, 96, 384, or 1536 wells may be necessary. However, for 3D cell imaging, these microplates may not be ideal due to the geometric shape and construction materials with some types having thick well bottoms that could reflect and reduce light penetration, or interfere and scatter light from detection on the microscope system (Auld *et al*. 2020). The use of specialized optical microplates consisting of glass or quartz are ideal materials for best performance optics, but are more expensive as compared to plastic counterparts and require enhanced ECM or other surface substrate coatings such as poly-lysine, a synthetic positively charged polymer to aid in cell attachment. Unpublished observations from HCS practitioners that mention glass surfaces still may not translate to the same biological and morphological representation as found in tissue culture treatment plastic microplates or dishes. However, traditional plastic polymer materials used in microplate fabrication are not necessarily ideal for optics, and molding process can increase variability from well-to-well locations across the microplate. More recent advancements in copolymer materials technology, such as cyclic olefin (Niles and Coassin 2008), have improved optical performance while maintaining well bottom thicknesses approaching a desirable microscope coverslip #1.5 (0.17 mm) that closely matches microscope objective lens requirements for optimal resolution (Piston 1998). These microplates are typically flat bottom and therefore are excellent for improved light penetration across the well surface but lack any artificial scaffold or matrix to support 3D cell models. A common practice when using these flat bottom microplates is pre-coating or seeding cells with agarose, hydrogel, Matrigel, or other similar matrix materials as shown with alginate to promote generation of 3D architecture (Tibbitt and Anseth 2009; Cavo *et al*. 2018).

Another more practical and simple approach for first-time introduction to 3D cell models or even for experienced HCS practitioners in screening is the use of ultralow attachment (ULA) microplates. These microplates directly depend on the geometric structure of a conical-like funnel shape to force cells to a small area at the bottom of well, thus promoting self-aggregation of cells using natural gravity to form spheroid-like structures over 24 to 96 h. One of the challenges with the ULA microplates for HCS imaging and microscopy is the geometric curve that reflects light differently than a typical flat bottom microplate. Ideally, the spheroid needs to be properly centered in the bottom of the well for robust imaging. In most cases, low magnification objective lens such a × 5 or × 10 objective lens is used when long working distances are required such as microplates with thick bottom thicknesses or when a spheroid is within or on top of an artificial matrix. In some cases, the use of a × 20 objective lens can be used for imaging spheroids in a ULA microplate; however, with larger objective lenses, there are limitations related to the spheroid size that could be outside of the detection pixel array of the camera chip requiring montage (side by side) imaging that requires longer acquisition times and the potential for out of focus images in the curvature in ULA microplate wells. Other methods of force assemble of 3D cell organoid cell models can be achieved by using magnetic nanoparticles previously discussed; the spheroids are formed within hours that then can be readily imaged with most HCS imaging and microscope systems. Regardless of the model, the spheroid cell structures do not always localize in the centroid area of the well across the microplate making them less uniformed during image acquisition.

Imaging 3D cell models in these different supporting environments creates variables that need to be recognized to formulate strategies for efficient image acquisition. Foremost, 3D cell colonies and individual spheroids are typically heterogeneous in size, shape, and location within the artificial environment with and without matrices. This is described in detail by Kenny and colleagues (Kenny *et al*. 2007) where they found that the morphology of the 3D clusters also differs from heterogeneity of the model itself, finding that cancer models may exhibit well-organized cell structures from typical cells and disorganized structures that depicts cancer cells with multiple nuclei and amorphous appearance in atypical cells. The heterogeneity is further supported by Roerink and colleagues where they investigated the diversification of intra-tumor phenotypes with corresponding genotypes in colorectal cancer cells (Roerink *et al*. 2018), thus providing evidence of the importance of single-cell and intra-well identification of individual tumoroid cluster phenotypic characteristics by 3D image analysis.

Although not necessarily considered high throughput, a review on the organ-on-a-chip (OOC) cell model devices showcases several commercially available systems (Zhang and Radisic 2017), many of which have been adopted and are amenable for HCS image acquisition and analysis. Examples of HCS imaging in these OOC models include the HepatoPac model (Trask Jr. *et al*. 2014), and more recently, researchers at AstraZeneca (Peel *et al*. 2019) adapted confocal HCS imaging with organ-on-a-chip model from Emulate containing primary human hepatocytes. One of the reasons for the recent surge of using HCS imaging instruments for such studies is greater accessibility to these devices in laboratories and understanding the advantage of speed to capture images in a non-bias automated fashion over traditional microscopy systems.

When interrogating 3D cell model systems, the microscope optical light excitation and emission from fluorescent biomarker probes plays a critical role in light absorption, penetration, scatter, and transmission to detect and resolve regions of interest within the cell. These combined attributes are often referred to the physics of the Beers-Lambert Law that is best described by Swinehart (1962) that concludes in simplest terms that transmission of light is dependent on concentration and thickness of the media and the attenuation of light scatter. In context of HCS imaging, confocal systems are the ideal HCS imaging tool for acquiring 3D cell model systems to remove out of focus light; reduce light scatter; and improve overall light penetration, speed, and sensitivity. The HCS confocal imagers typically are configured with laser or LED light sources and use spinning disk technology to reduce photobleaching, phototoxicity, and increase overall image acquisition speeds.

### Reagents and probes for 3D cell imaging

The use of reagents and biomarker probes including fluorescent protein reporters that are known to work well in 2D cell monolayer models may or may not exhibit the same protein expression or performance in 3D cell model systems due to physical barriers, oxygen content and nutrient levels within the core of a spheroid, and other environmental conditions (Edmondson *et al*. 2014; Wenzel *et al*. 2014; Grist *et al*. 2019). Therefore, HCS practitioners need to review and verify these potential caveats during assay development and validation experiments before proceeding. Protein expression measured by fluorescent protein reporters is perhaps the easiest transition from a 2D cell model to a 3D cell model since very little manipulation is required other than providing an appropriate environment to promote 3D cell growth. The LaBarbera laboratory demonstrated the use of a dual EGFP and mCherry fluorescent protein reporter system to measure epithelial–mesenchymal transition (EMT) biology using flat bottom microplates containing agarose and Matrigel matrices, but also used additional commercially available fluorescent bioprobes Hoechst 33342 and DRAQ7™ to label the nucleus for object identification and cell membrane permeability respectively in HCT116 colorectal cancer cell line (Trask *et al*. 2018). This added benefit boosted the multiplex capability of the 3D cell model to gain new insights to address important questions about the biological response in 3D cell model system, such as location of nucleus, nuclear morphology, toxicity, and existence of a necrotic core as evident by the accumulation of the DRAQ7 intensities. Additionally, sometimes not considered, the use of reference control compounds or other chemicals that exhibit fluorescence can be used as tools to measure the penetration into the 3D cell model system. Shan *et al*. (2018) revealed four anthracycline compounds (doxorubicin, daunorubicin, epirubicin, and mitoxantrone) that exhibited fluorescent properties as useful tools in HCS imaging to determine distribution and penetration into a 3D multicellular tumor spheroid model in ULA microplates.

**Figure 8.**
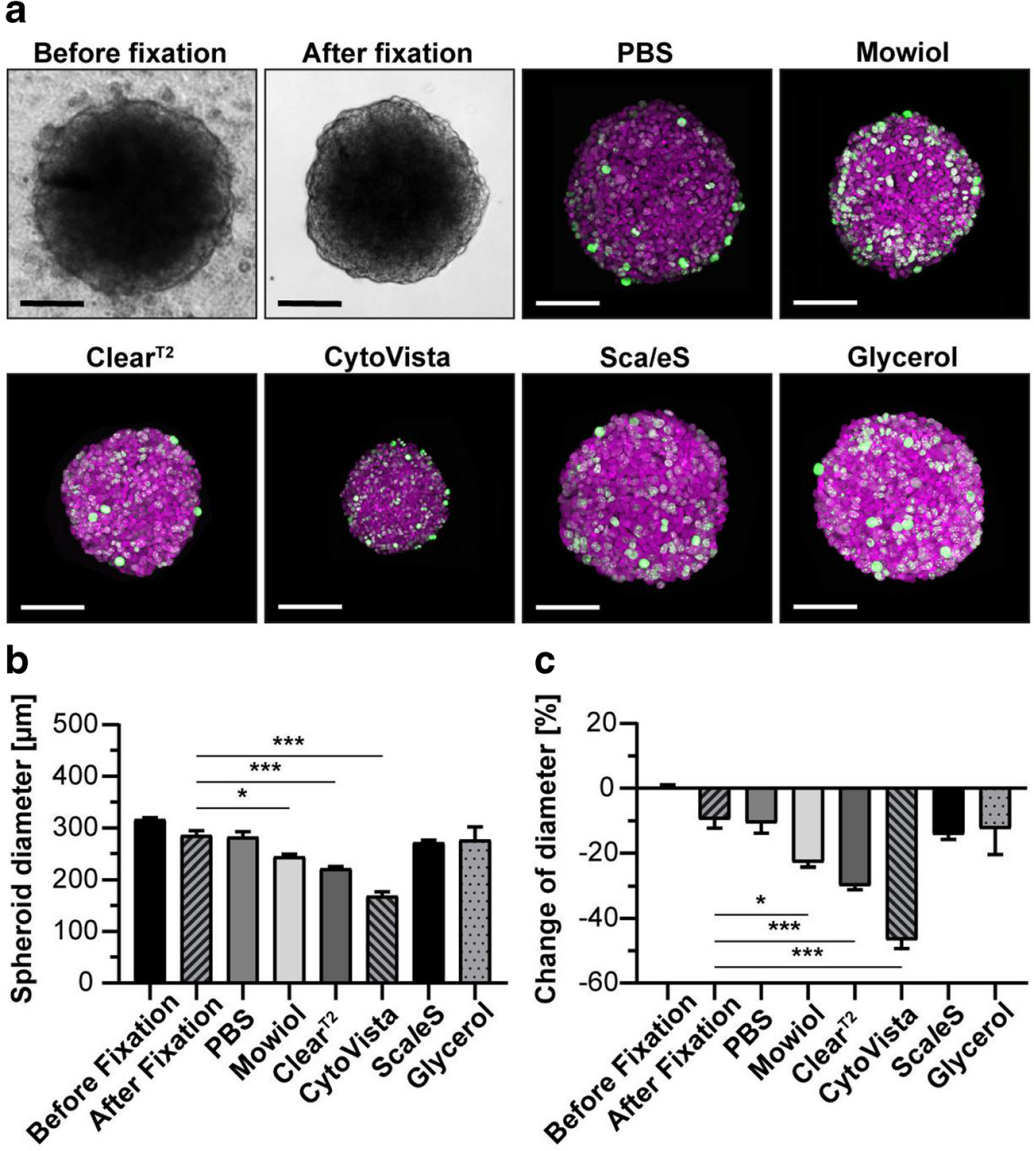
Impact of clearing methods. HaCaT keratinocytes were fixed and labeled with anti-KI67 antibody (*green*) and DRAQ5 (*magenta*), followed by clearing. Images captured on Leica TCS SP8 confocal microscope (Leica Microsystems). (*a*) Bright-field spheroids image stack before and after fixation to establish baselines and fluorescent confocal image stack for each clearing method to measure changes. (*b*) Average spheroid diameter for each clearing condition. (*c*) Spheroid diameter change relative to pre-fixation. Graphs shows mean+ standard deviation (SD); *n* ≥ 9; *p ≤ 0.05, ***p ≤ 0.001. Figure reprinted with permission and courtesy of Nurnberg et al. 2020.

The combined use of aldehyde or alcohol fixation in endpoint assays with clearing agents to strip lipids and proteins can help reduce light scatter and improve light penetration into the 3D cell models which may permit full volumetric imaging of the entire cell model (Boutin and Hoffman-Kim 2015; Boutin *et al*. 2018). A process called fast optical clearing method by Zhu and colleagues is promising with claims of nearly a 2-min incubation resulting in 86% fluorescent stability for up to 11 days (Zhu *et al*. 2019). More recently, a study of six different clearing approaches for 3D imaging demonstrated that the combination of paraformaldehyde fixation followed by 88% glycerol immersion was among the best approaches without significantly altering cell morphology (Nurnberg *et al*. 2020) (see Fig. 8). However, the same study demonstrated the CytoVista reagent did significantly alter cell morphology by shrinkage, which supports a previous study by Richardson and Lichtman, verifying solvent based agents including benzylalcohol has a detrimental effect on cell morphology. While the depth penetration detection of bioprobe markers in 3D cell models varies in live cells, fixed cells, or fixed cells that undergo an additional clearing step, these methodologies each have advantages and disadvantages for HCS imaging applications (see Table 2).

**Table 2:** Comparison and considerations for HCS imaging attributes in live cell, fixed cell, and fixed cells plus clearing processing

	Live cell	Fixed	Fixed + clearing
Light penetration	~ 50–150 μm	~ 75–250 μm	> 200 μm
Kinetic imaging	Yes, real-time	Fixed endpoint only	Fixed endpoint only
Fluorescent biomarker probe	Higher background with lower SNR Subject to photobleaching and phototoxicity multiplexing is limited	Reduced background with higher SNR stable fluorescent intensity with reduced photobleaching multiplex 4 or more probes	Reduced background with higher SNR reduced fluorescent intensity and photobleaching multiplex; but dependent on clearing method
Cell morphology changes	No changes (ground truth)	Minor modification in alcohol-based fixatives	Slight to serve modifications with shrinkage or swelling
3D image segmentation	Outer surface; some resolved segmentation below spheroid surface; sample dependent	Outer surface and necrotic core is possible with thin sections < 200 u; sample and microscope dependent	Excellent for segmentation throughout sample; microscope dependent

### 3D image capture process

The cell model, reagents, probes, and vessels or microplates used are essential components for 3D imaging; but the instrument resolution requirements to make the biological measurements within a solid spheroid or cyst will help dictate the most efficient strategy for 3D image acquisition. A more common and laborious approach in traditional microscopy techniques to assess a single full 3D cell volume uses the practice of identifying two points, the bottom fluorescent surface (cells at the bottom of a slide or microplate well) and the top of the fluorescent surface or the highest penetration depth of detection in the 3D structure; both points exhibit distinct z-heights making up the detectable volume of the cell model. Since HCS imaging is an automated process, a much broader search range is typically required when interrogating 3D cell model systems with multiple clusters, colonies, or spheroids since the location and volume depth of each 3D cellular object are unknown at time of acquisition unless determined in advance. In some cases, the added benefit of automated intelligent image acquisition provides a method to automatically pre-scan microplate wells in high throughput to first identify the region of interest in XYZ dimensions using a low magnification objective lens, i.e., × 5, and then automatically rescan and refocus the same XYZ region of interest based on automated image analysis determinations with a higher resolution objective lens, such as a × 20 or greater, for improved resolution to address more in-depth biological questions. This process speeds up the overall image acquisition by limiting the number of fields not containing cells of interest in the well and at the same time reduces the overall number of required images to generate a full composition of the 3D architecture of the cell model. The objective lens used will determine the minimal z-stack slice sectioning, and this value is provided by the manufacturer’s recommendation. For example, a × 20 objective lens specifications may be 2 μm for each z-plane, whereas a × 40 objective lens may be 0.5 μm for each z-plane.

Oversampling may be a concern in 3D cell imaging and can create unnecessary data and additional time to acquire images. In practice, a series of z-stack slices are captured to encompass the entire 3D cell structure with the total stack equaling the volume of the visible structure; e.g., if an organoid is 100 μm thick, then a × 40 objective lens with a 0.5-μm z-plane would consist of 200 independent images for a single field for each fluorescent channel. The 3D image capture process is a critical step for accurate image analysis segmentation process; therefore, careful consideration about potential pitfalls needs to be addressed in advance (North 2006). The other often overlooked component of the 3D image acquisition process other than time is the number of images captured and data storage requirements that can easily generate gigabytes of data. For example, a 3D image stack from a single region of interest (one field) that is labeled with 3 different fluorescent wavelengths including a single transmission bright-field image having a tissue or spheroid that is 100-μm thick with an objective lens using 1-μm z-sectioning with a digital camera with 2000 × 2000 pixel array at × 1 binning would occupy less than 1.6 GB due to camera sensor saturation limits; when multiplying this number to include only 2 fields per well in a 384 microwell plate, the number swells to over 1 terabyte.

### 3D image visualization and analysis

**Figure 9.**
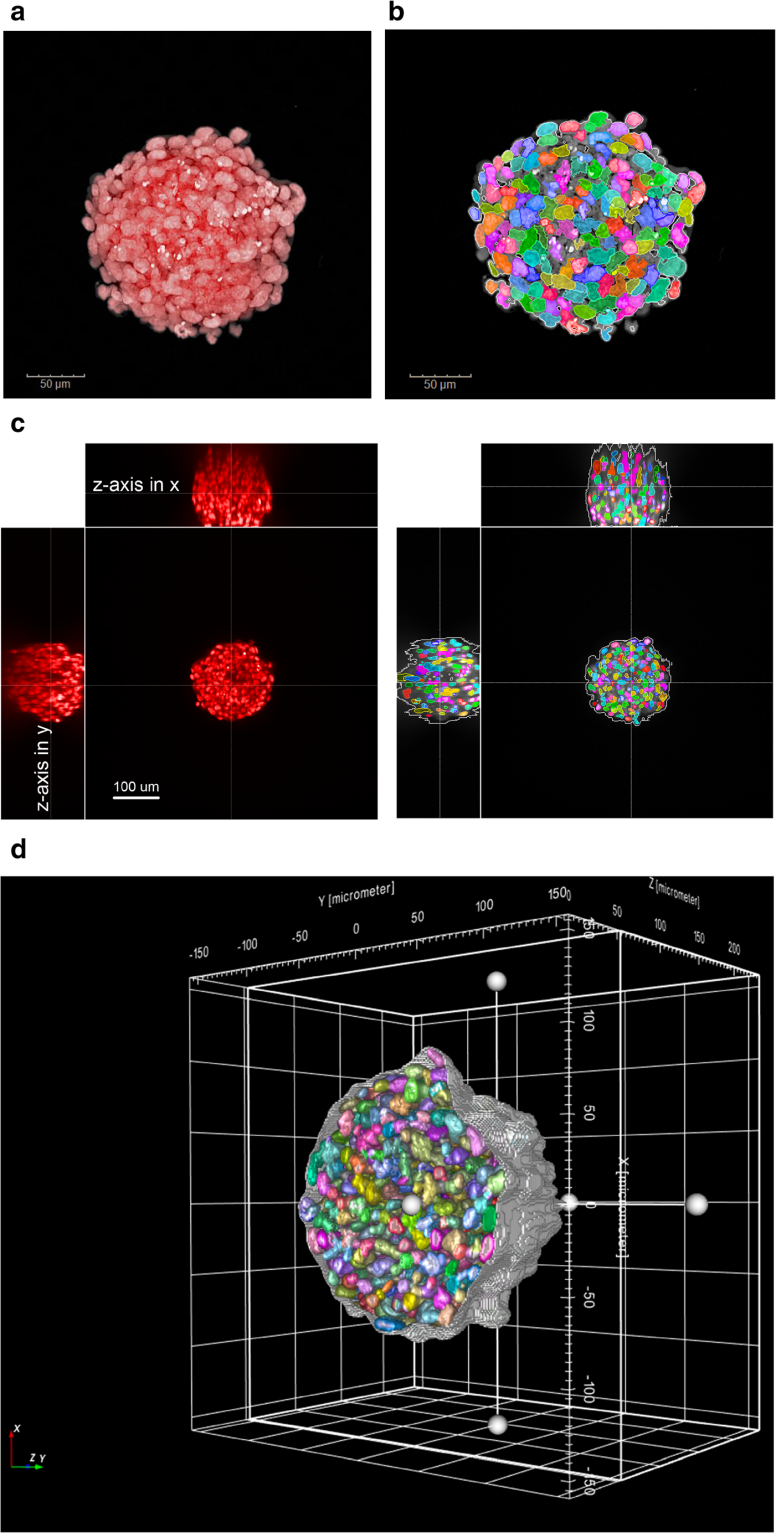
3D image analysis approaches. CHO spheroid labeled with DRAQ5 and captured with × 20/1.0 NA water lens on Opera Phenix HCS imager. (*a*) × 20 maximum intensity projection image. (*b*) 2D image analysis segmentation overlay of individual nuclei of maximum intensity projection image. (*c*) 3D reconstruction image projection of HeLa tumoroid labeled with DRAQ5; *z*-axis is observed in both *x*-axis and *y*-axis for each individual z-plane. (*d*) 3D image analysis segmentation overlay of individual nuclei through the entire volume.

Visual observation of the 3D images provides an overview of the expected outcomes of the labeling with probes, the imaging, and overall biology. However, visualizations alone is only considered a high level overview assessment about the quality of the image and is a biased interpretation of the biological response; therefore, quantitative image analysis is needed. The process of 3D image analysis is a daunting task that has a plethora of challenging factors (Long *et al.* 2012). There are many strategies to interrogate the 3D cell model using image analysis that first begins with 3D reconstruction of the cell model in XYZ dimensions allowing visualization in any direction from the top, bottom, left, right, or circular rotation (see Fig. 9). In some cases, a process known as deconvolution (Wiener 1964), which is an algorithm that uses point spread function or Fourier transformation to modify “blurred” images in “focused” images for improved visualization. However deconvolution comes at a cost of both time consumption and difficulty to reproduce for quantitative 3D segmentation.

**Figure 10.**
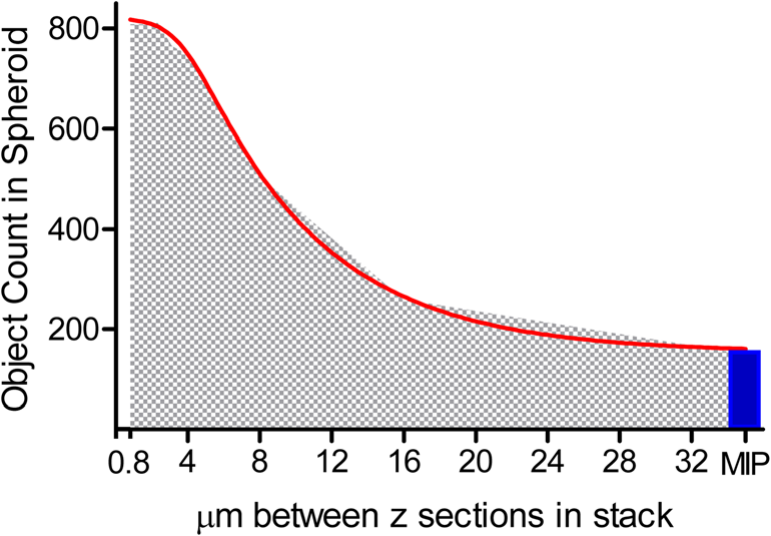
The quantitative image analysis segmentation of CHO spheroid illustrates missed nuclei counts when z-slice intervals increase in 3D volumetric image analysis segmentation or when compared to 2D image analysis of maximum intensity projection (MIP); spheroid image includes 301 individual slices at a minimum of 0.8-μm interval spacing from a 20xW lens. *X*-axis represents micron spacing between z-stack starting at 0.8 μm and doubling up to 32-μm spacing per slice; *y*-axis indicates the number of volumetric nuclei object count in the 3D sphere. Nonlinear regression asymmetric 5-parameter curve fit *R*^2^ is 0.9993 with EC_50_ (1/2 maximum) of 8.798-μm z-interval spacing; graph and data generated in GrapPad Prism.

There are three commonly used HCS imaging processes to analyze 3D image slices or stacks: (1) maximum intensity projection; (2) slice by slice of z-stack image analysis; and (3) volumetric image analysis where each pixel in volume, called voxel, is measured in context of the entire 3D object. Foremost, maximum intensity projection, initially developed for nuclear medical images (Wallis *et al*. 1989), is the simplest and most often used methodology to analyze 3D models in HCS imaging. The method renders or collapses all images captured from the 3D stack into a single 2D image display allowing segmentation with traditional 2D image analysis algorithms. The advantage of this image analysis method is speed and use of existing algorithm solutions; however, in the context of the 3D cell models, the architecture is lost and 3D depth and spatial relationships are removed as all information is merged into a single orthographic image. The slice by slice image analysis uses traditional 2D image analysis algorithm to analyze individual slices in the stack (see Fig. 10). Its advantage is the ability to measure through the entire 3D image stack, but the disadvantage is the process that requires more time since every image needs to be processed, spatial information in context of the full 3D cell model is lost, and there is a possibility of counting cellular objects that are split between planes multiple times that could modify quantitative analysis. Volumetric image analysis is the most comprehensive method that provides full contextual measurements of the 3D cell model with spatial information and relationships within the 3D environmental space in all directions (XYZ), but it is more computer CPU or GPU processor intensive and therefore slower to generate data.

A caveat for measuring in vitro 3D structures in basic research is that there are no uniform or standardized 3D image analysis segmentation programs available across the wider scientific community, making interpretation of the data somewhat ambiguous when comparing and contrasting quantitative image analysis data without benchmarking to a ground truth analysis. Additionally, in all approaches discussed, user bias can alter the end results from selecting only certain images and setting manual thresholds during the image analysis setup process, e.g., determining background over fluorescence. A further challenge in 3D image analysis is not all 3D cell structures, spheroids, or organoids are equal in size and intensity, even within a single well, making the overall statistical measurements from the heterogeneous response more variable. To help circumvent some of this bias and challenges, the introduction of machine learning to reduce practitioner’s intervention allows an artificial intelligence approach to determine the end result; but this needs to perform on a known uniform training set to improve the overall confidence, provide a reduction in the variability of the data, and increase reproducibility of the experimental assay (Scheeder *et al*. 2018). In some cases, the use of deep learning algorithms may be employed to train image sets to use a segmentation-free approach to improve the overall data. Regardless of the methodology and approach of machine learning or manual biased threshold setting, all of the data needs to be validated by running multiple replicate conditions to undoubtedly demonstrate the robustness and reproducibility of scientific data.

## Conclusion

The ongoing development of 3D culture models continues to evolve and provide researchers with more physiologically relevant representations of in vivo biology; however, the markers used to indicate change can be difficult to measure in larger and more complex 3D samples compared to using monolayers of cells. The advantages and limitations of the combination of the 3D culture model system and the assay to interrogate the desired markers need to be considered together to choose the best “fit-for-purpose” assay. Limitations of biochemical assay reagents and the physics of light penetration for microscopic imaging must be considered. Building awareness of the factors to consider when choosing an assay to interrogate 3D culture models will help generate data with fewer artifacts and greater reproducibility.
